# Supplementary material for: Identification and Characterization of TMEM119-Positive Cells in the Postnatal and Adult Murine Cochlea
Source: Brain Sci. 2023 Mar 20;13(3):516. doi: 10.3390/brainsci13030516 (PMC10046579; doi:10.3390/brainsci13030516)
Supplement: Supplementary file 1 [file brainsci-13-00516-s001.zip › brainsci-2196763-supplementary.pdf]

Supplementary Materials

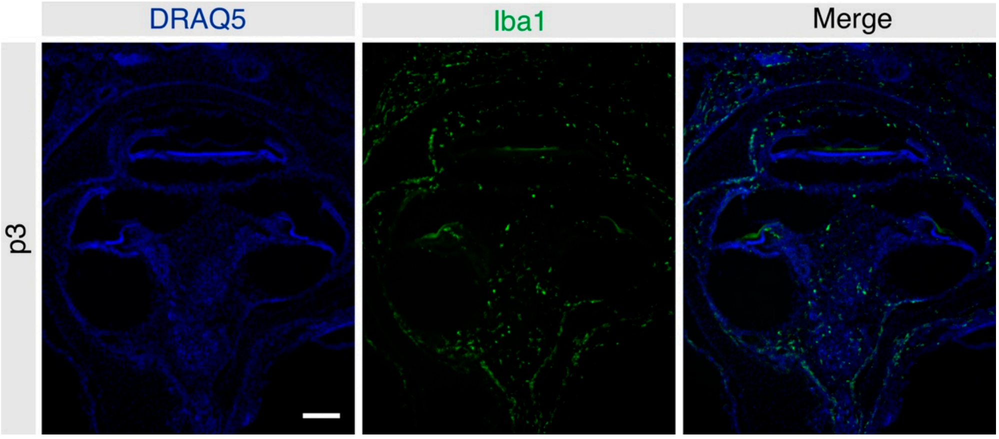

Figure S1. Frozen sections of the immature mouse cochlea at p3 stained for Iba1 (green). Nuclei were counterstained with DRAQ5-labeling (blue). Iba1 expression was observed in scattered cells with dendritic morphology across different cochlear regions at p3. Scale bar: 200 μm.
